# Ionic surfactants alter virus surface properties and electrostatic interactions in aqueous systems

**DOI:** 10.1093/femsmc/xtaf011

**Published:** 2025-09-11

**Authors:** Makayla Loey, Gabriel Costa Alverni Da Hora, Jennifer Weidhaas

**Affiliations:** University of Utah, Department of Civil and Environmental Engineering, 110 Central Campus Drive, Suite 2000, Salt Lake City UT 84112, United States; University of Utah, Department of Chemistry, 315 1400 E, Salt Lake City UT 84112, United States; University of Utah, Department of Civil and Environmental Engineering, 110 Central Campus Drive, Suite 2000, Salt Lake City UT 84112, United States

**Keywords:** enveloped and non-enveloped viruses, isoelectric point, minimum inhibitory concentration, surfactants, molecular dynamic simulations, virus hydrated diameter

## Abstract

Interactions between viruses and sub-inhibitory concentrations of surfactants in water systems are understudied. At concentrations below the minimum inhibitory concentration (MIC), surfactants may interact with virus surface proteins without virus inactivation and alter virus surface properties. This study determined the MIC of benzyldimethyldodecylammonium chloride (BAC) and sodium dodecyl sulfate (SDS) on human adenovirus (ADV, non-enveloped, dsDNA) and mouse hepatitis virus (MHV, enveloped, ssRNA), and how sub-MIC surfactants influence virus isoelectric point (IEP), hydrated diameter, and interact with virus surface proteins. Both surfactants had MICs of 1 mg/L over 60 minutes. Experimental IEPs were lower than IEPs estimated based on amino acid structures. The ADV IEP was 3.8 without surfactants and dropped to 3.3 with BAC and lower than 3 with SDS. The MHV IEP was 4.2 without surfactants and decreased to 4.1 with SDS and 3.4 with BAC. Dynamic light scattering showed SDS and BAC decreased ADV hydrated diameter from 142 ± 8 nm (no surfactant) to 109–116 nm, while MHV decreased from 150 ± 10 nm (no surfactants) to 132–140 nm upon surfactant exposure. Molecular dynamics simulations revealed that SDS, due to its multivalent sulfate headgroup, forms numerous intimate contacts with the MHV spike protein that markedly perturb its electrostatic environment. In contrast, BAC engages only sporadically and diffusely with the protein, indicating a much weaker influence on its structure and electrostatics. Overall, this study showed that ionic surfactants can influence virus properties thus altering virus interactions with surfaces in engineered and natural systems.

## Highlights

- Ionic surfactants alter enveloped and non-enveloped viruses surface properties.
- Minimum inhibitory concentration of ionic surfactants towards both enveloped and nonenveloped viruses is 1 mg/l within 60 minutes.
- Modeled virus surface proteins IEP is higher than experimentally determined IEP
- Virus zeta-potential and hydrated radii both were influenced by ionic surfactants.
- MD simulations: SDS forms stronger, localized interactions with viral proteins; BAC exhibits a slightly weaker, diffuse binding.

## Introduction

The utilization of surfactants, also referred to as surface-active agents, is pervasive in industrial applications to produce detergents, paints, polymers, textiles, pharmaceuticals, pesticides, paper, and personal care products (Badmus et al. 2021). These amphipathic molecules containing dual hydrophilic heads and hydrophobic tails are classified by their electrical charge on the hydrophilic portion, which can be divided into anionic, cationic, non-ionic, and amphoteric categories (Glassman 1948). Concomitant with the industry advancement and increase in material consumption, there has been a notable surge in the utilization of surfactants. It is noteworthy that the annual synthetic surfactant production is approximately 7.2 million tons (Najim et al. 2022). The surfactant global market has an anticipated growth rate of 20% by 2025 (Badmus et al. 2021). Given the widespread use of surfactants, it has been reported that surfactants present in domestic wastewater can range from 1-10 mg/L, while wastewater from the surfactants manufacturing industry can contain up to 300 mg/L (Zhang et al. 1999). The research indicates that most secondary effluents from wastewater treatment plants contain 1-3 mg/L surfactants (Giger et al. 1989, Bautista-Toledo et al. 2014).

In addition to surfactants being present in wastewater, viruses are also ubiquitous. Treating or inactivating viruses are of utmost concern for the design of water reuse systems and for drinking water systems obtaining water from indirect potable reuse sources. Membrane filtration and disinfection are commonly applied to remove or inactivate viruses in water and wastewater. Ultrafiltration membranes with a nominal cutoff size of 0.1 um or 100 nm, and nanofiltration membrane with a nominal cutoff size of 0.001 um or 1 nm, are suitable for filtering waterborne viruses (Leibowitz et al. 2011). However, viruses often aggregate or behave like colloids (i.e. electrostatic interactions with other surfaces are important) in aqueous environments, allowing them to be removed even with membrane filters that have larger nominal pore sizes than the effective diameters of viruses, such as microfiltration membrane (Jin and Flury 2002, Sinclair et al. 2018, Zhdanov 2021). Therefore, either the membrane surface or virus charge may significantly influence virus rejection by membranes. Conversely, ionic surfactants in water and wastewater may alter virus surface charges sufficiently to impact the efficacy of virus rejection via electrostatic interactions or treatment by other technologies (Sinclair et al. 2018). Therefore, there is a need to understand surfactant and virus interactions, especially as they may impact water quality.

Viruses are either enveloped or non-enveloped (Corpuz et al. 2020). Both virus types contain a viral genome (DNA or RNA) and a protein capsid surrounding the genome, which serves to protect the genome from the environment (Louten 2016). Enveloped viruses will possess an additional lipid bilayer membrane that encloses the capsid protein and may contain spike proteins inlaid on the membrane. This lipid bilayer membrane is unstable and will interact with host cells during viral infection, facilitating the exchange of information with host cells (Flint and Shenk 1997, Bárcena et al. 2009, Heffron and Mayer 2021). Non-enveloped viruses lack this lipid bilayer membrane enclosing the capsid (Louten 2016). These structures, the envelop membrane and the capsid, have been previously investigated to a limited extent with respect to their interaction with surfactants.

**Figure 1.**
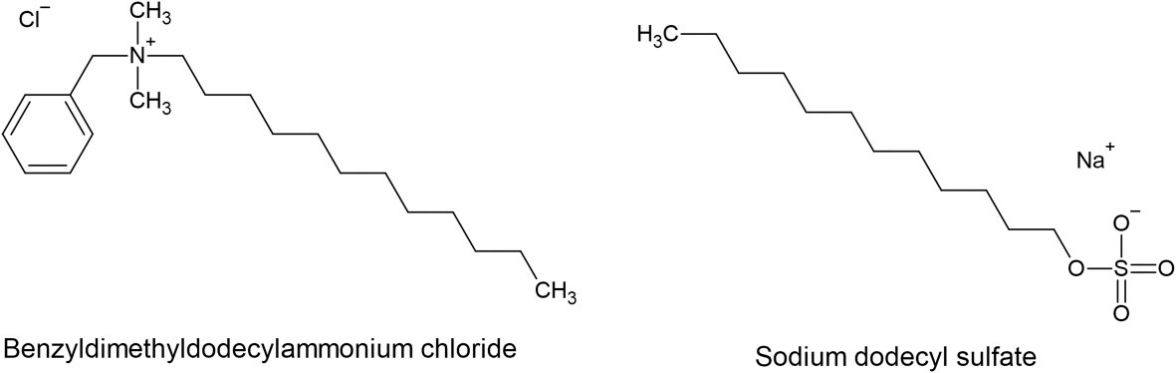
Anionic surfactant Sodium Dodecyl Sulfate (SDS) and cationic surfactant Benzalkonium Chloride (BAC) structure by ChemDraw (Perkinelmer, Waltham, Massachusetts, US).

The scientific literature has primarily focused on virus inactivation by surfactants, with fewer studies investigating the underlying mechanisms of interaction. Early studies established that the dissociation of virus membrane proteins with surfactants was proportional to temperature and surfactant concentration (Becker et al. 1975). Anionic surfactants such as sodium dodecyl sulfate (SDS, Fig. 1), have been reported to denature and unfold both monomeric and subunit proteins of viruses (Flint and Shenk 1997). The SDS denaturing protein capability likely explains the previously reported virus inactivation of both enveloped (herpes simplex type 2) and non-enveloped (papillomaviruses) viruses in the presence of this surfactant (Flint and Shenk 1997). More recent investigations have shown that the addition of SDS to goat milk can effectively inactivate human immunodeficiency virus (HIV) and caprine lentivirus in milk (de Sousa et al. 2019). To elucidate the interplay mechanisms between SDS and viruses, a study using influenza viruses and SDS using isothermal titration calorimetry (ITC) revealed a negative enthalpy change when 5 uL of 1.5 mg/mL purified virus protein was injected every 14 seconds to ITC cell with 0.175 mmol/L of surfactant solution (Kawahara et al. 2018). This finding indicates that the electrical interaction between influenza viruses and SDS was endothermic (Kawahara et al. 2018). The cationic surfactant benzyldimethyldodecylammonium chloride (BAC, Fig. 1) is a biocide that has been reported to disrupt viral membrane charge distribution in the membrane bilayers via the charged nitrogen group in the BAC alkyl chain (Merchel Piovesan Pereira and Tagkopoulos 2019). Further, BAC has been reported in a recent study to alter microbial membrane composition and modify the efflux pump to decrease susceptibility to the surfactant (Merchel Piovesan Pereira and Tagkopoulos 2019).

Surfactant concentrations used as a disinfectant for virus inactivation are significantly higher than those expected in a water treatment system. While the reported concentration of surfactants in wastewater ranges from 1 to 3 mg/L (Giger et al. 1989, Bautista-Toledo et al. 2014), the concentrations used for virus disinfection are 3 to 10 times higher and surfactant disinfection effectiveness may vary by virus serotype. For example, incubation at 37 °C for 1 hr with as little as 0.125 mg/L of BAC reduced ADV serotype 20 virus infectivity by 3.0 log_10_ (Bélec et al. 2000). In contrast, for ADV serotype 5, between 30 and 100 mg/L of BAC was reported to decrease viral titer by more than 1 log_10_ over 1 hr at 33 °C (Merchel Piovesan Pereira and Tagkopoulos 2019). When eggs were soaked in 2% SDS and 5% levulinic acid for 1 minute, influenza A H3N2 was reduced by 2.23 log plaque forming units (PFU) (Aydin et al. 2013).

**Figure 2.**
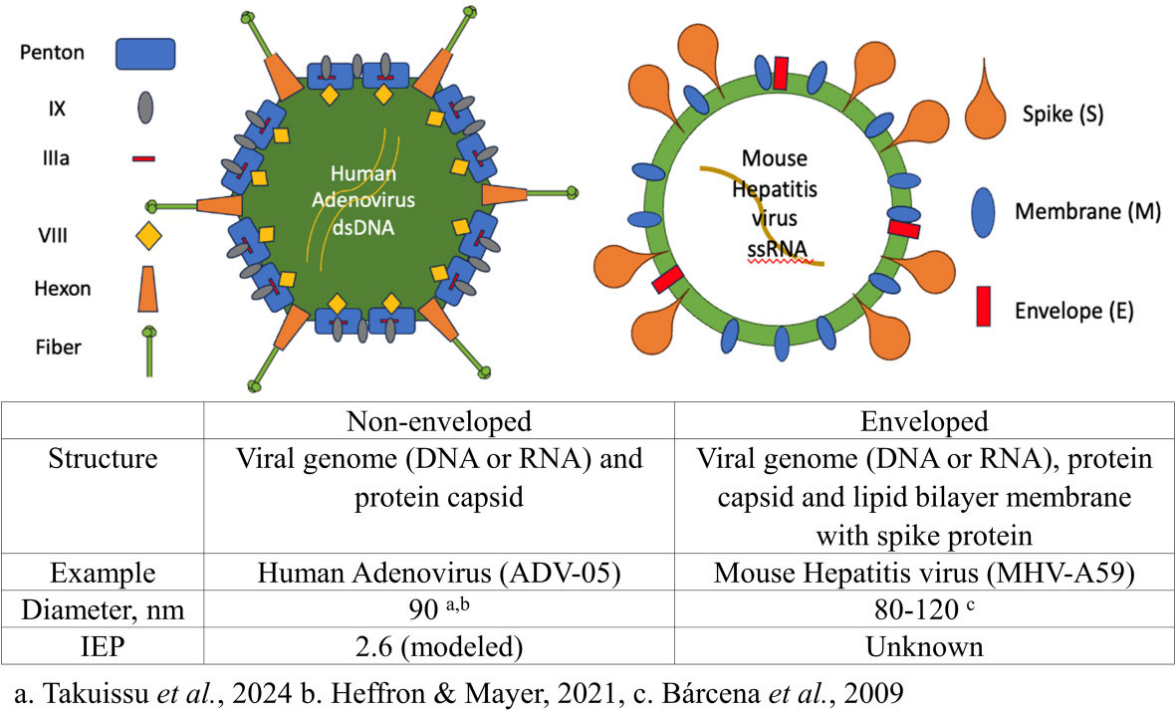
Structures of ADV and MVH, literature reported virus diameter and empirical IEP.

Given that surfactants in wastewater are at concentrations lower than those expected to inactive some viruses, it is necessary to explore surfactants’ minimum inhibitory concentration (MIC), which is the lowest concentration of surfactants required to inhibit viral replication. Specifically, it is crucial to examine the impact of surfactants on virus membranes and surface proteins at concentrations lower than the surfactant MIC. Further, at these low surfactant concentrations found in wastewater at which viruses are still infective, studies are needed to understand how the virus surface properties change as a function of interactions with surfactants and thus may influence water treatment technology efficacy and interactions with various surfaces. It is likely that at low concentrations, the ionic surfactants may change the virus isoelectric point (IEP), or the pH at which the overall charge of the viral particle is neutral. If the viral surface charge changes as a function of surfactant interactions, electrosteric interactions with charged surfaces found in hospital settings, in water treatment systems, and in the environment may be impacted. To bridge this knowledge gap, this study reports on the interaction of the enveloped virus murine hepatitis virus strain 59 (MHV-A59, hereafter MHV) and the non-enveloped virus human adenovirus serotype 5 (AdV-05, hereafter ADV) (see Fig. 2), with ionic surfactants. Both the anionic SDS and cationic surfactant BAC will be used to represent distinct categories of surfactants. The result will provide insights into the effect of ionic surfactants on viruses, especially below the MIC, contributing to an understanding of viruses’ property change in the context of water and wastewater treatment processes.

## Materials and methods

### Cell culture and virus propagation

Cell cultures used to propagate the viruses were HEK-293 (human kidney epithelial cells) and L929 (mouse fibroblast connective tissue cells) for propagating ADV and MHV, respectively. The cell lines were purchased from the American Types Culture Collection (ATCC, Manassas, Virginia, US). Both MHV and ADV were obtained from ATCC (Manassas, Virginia, US). Cultivation of cell lines occurred in 75 cm^2^ U-shaped vented flasks (Sigma-Aldrich, St. Louis, Missouri, US) containing 13.5 ml of Eagle's Minimum Essential Medium (EMEM) (ATCC) and 1.5 ml of either fetal bovine serum (FBS) for ADV or horse serum (HS) for MHV (MP Biomedical, Inc, Santa Ana, California, US). The medium-to-serum ratio was maintained at 9:1 (v: v). Cell sub-culturing occurred when 90% cell confluence was observed via inverted microscopy (MU 900 AmScope, Irvine, California, US). Cells were collected by utilizing trypsinization (3 ml of 0.25X trypsin [ATCC] used for a 75 cm^2^ flask) for seven minutes to disassociate cells from the flask surface. Trypsin was then neutralized by adding 7 ml of cell medium. The disassociated cells were collected and centrifuged at 1500 rpm for five minutes, and the supernatant was removed by pipette. Cell pellets were then re-suspended by adding 5 ml of new medium with serum and injected into five new flasks before incubating at 37°C in a 5.5% CO_2_ atmosphere (VWR, Radnor, Pennsylvania, US). Cells were stored in liquid nitrogen after preparation according to the methods published previously (Shuipys & Montazeri 2022) with the following exceptions. Prior to freezing the cell pellets were re-suspended in a Recovery Cell Culture Freezing Medium (Fisher Scientific, Waltham, Massachusetts, US). A 2-CHIP cell counting plate (Bulldog-Bio, Portsmouth, New Hampshire, US) ensured the cell counts reached 10^6^ cells in 1 ml before storage. Mr. Frosty^TM^ Freezing Container (Thermo-Fisher Scientific, Waltham, Massachusetts, US) filled with isopropanol (Sigma-Aldrich) was used to facilitate the cell cooling process in a -20 °C laboratory standard freezer (VWR) before transferring the container to a -80 °C freezer (Thermo Scientific, Waltham, Massachusetts, US) for an additional 24 hr to achieve an ideal rate of cooling. Finally, cells in the Nalgene cryogenic vial (Sigma-Aldrich) were transferred to a liquid nitrogen tank for storage.

For virus propagation, when the L929 cell reached 90% confluence, 3 ml of existing MHV in EMEM solution was added to a 75 cm^2^ flask and incubated at 37 °C in 5.5% CO_2_ for 60 min. To enhance virus inoculation, the flask underwent periodic, gentle agitation every 15 min. Then 22 mL of a 9:1 volumetric ratio of NCTC-135 virus growth medium (Sigma-Aldrich) and horse serum were added to the flask to assist virus propagation. The incubation period to facilitate MHV virus replication spanned a duration of 18–24 hr before harvesting. Adenovirus cultivation deviated slightly from the MHV cultivation in that EMEM supplemented with 2% FBS was used, and the virus infection duration was extended to 2 hrs. The post-infection incubation period was prolonged to 48 hr. Upon completion of virus harvesting, all solutions were transferred to a 50 ml centrifuge tube (Corning, Corning, New York, US) and frozen at –80 °C for 20-30 min, followed by thawing to optimize intracellular virus particle release. Subsequently, centrifugation at 3,000 rpm for 10 min (5910R centrifuge Effendorf, Hamberg, Germany) was employed to precipitate all cell debris and the supernatant virus solution was collected for further virus purification.

### Virus purification

Virus purification was used to concentrate the original virus stock solution to a higher titer. The starting and final virus purified concentrations were determined using an 8 fold dilution series of 40 ul of each virus added to the appropriate cell lines at 90% confluence in duplicate wells (Falcon^TM^ 96-Well flat bottom microplate, Fisher scientific, Waltham, Massachusetts, US). The cells and viruses were then incubated for 18 hr for MHV and 48 hr for ADV. The viral infectivity was confirmed by inverted microscopy and viral titer was determined by TCID_50_/ml calculator.

Virus purification was conducted by employing a 20% sucrose solution (Sigma Aldrich) in de-ionized water. In each ultracentrifugation tube (Seton Scientific, Petaluma, California, US) 8 ml of viral solution was first placed into the tube and then 1 ml of 20% sucrose solution was injected into the bottom of each tube using a long-tip pipette. Then the 8 ml virus sample was centrifuged in a swinging bucket rotor ultracentrifuge (L8-70 M, Beckman, Brea, California, US) at 20,000 rpm (70 000 xg) for 2 hr. After centrifugation, the supernatant was removed, and the centrifuge tube was dried using sterile q-tips (Medline, Northfield, Illinois, US). Then 100 uL of 1X PBS buffer at pH 7.2 was added to each centrifuge tube and the tubes were stored at 4 °C overnight. The next day the purified virus would be visible as a white pellet at the bottom of the centrifuge tube. The virus pellet would then be resuspended in 1X phosphate buffer saline (PBS) and stored at either 4 °C for immediate use or at –80 °C for future use.

### Virus zeta-potential estimation and isoelectric point determination

A V7 zeta-potential analyzer (Dynamics, New Orleans, Louisiana, US) equipped with both dynamic lights scattering (DLS) and phase analysis light scattering (PALS) features was employed to determine the zeta potential of the viruses with and without exposure to surfactants and the virus particle hydrated radii. The determination of the virus particle zeta potential was conducted by employing a Smoluchoski model based on the particle size and ionic strength. To determine the IEP of ADV and MHV, a 0.1 M ionic strength buffer solution from pH 3 to 9 was prepared as solvent. The pH was maintained using either a citric buffer (citric acid/sodium citrate) for pH 3, 4, and 5; a phosphate buffer (phosphate monobasic/phosphate dibasic) for pH 6 and 7; or a borate buffer (borate acid/sodium hydroxide) for pH 8 and 9. Ionic surfactants were then introduced to each of the buffer solutions to reach concentrations of either 5 or 10 mg/L. Subsequently, 30 purified MHV viruses with a concentration of 10^5^ TCID _50_/ml or ADV viruses with a concentration of 10^7^ TCID _50_/ml were introduced into 1.5 ml surfactant-buffer solution, followed by sonication for 10 seconds to prevent virus aggregation. Zeta-potential of 40 ul of each mixed solution was then determined. Controls included viruses in buffer solution without surfactants.

In addition to the experimentally determined IEP, two bioinformatic tools, Expasy (Artimo et al. 2012) and EMBOSS (Rice et al. 2000) were also chosen to calculate the IEP for the surface proteins of both viruses at different pH in the absence of surfactants. For ADV, three major surface proteins (hexon, fiber, and penton) and three minor surface proteins (IX, IIIa, and VIII) were modeled. For MHV, three major surface proteins were modeled, including the membrane protein (M), enveloped small membrane protein (E), and spike protein (S). The protein sequences were collected from UniProt (Consortium 2019).

### Surfactant minimum inhibitory concentration to viruses

The minimum inhibitory concentration (MIC) is defined as the lowest concentration of an agent that can completely prevent proliferation of the tested organism (Kowalska-krochmal and Dudek-Wicher 2021). In this research, the MIC of SDS and BAC on the infectivity of MHV and ADV was determined by a viral dilution, propagation in cells, and quantification by qPCR. Further, the MIC of the surfactants to the cell lines HEK-293 and L929 was determined prior to testing for virus MIC by the dilution method.

Prior to determining the MIC for surfactants on ADV and MHV, two control studies were conducted. In the first control study the toxicity of SDS and BAC to the cell lines HEK-293 and L292 was evaluated by using propidium iodine (PI) dye (2 uM, Sigma-Aldrich) as a cell live/dead indicator. A cell toxicity study was conducted where surfactants at concentrations of 0, 2, 4, 6, 8, 10 and 12 mg/L were added to 150 ul of complete cell media containing PI. This surfactant-cell media-PI preparation was added to a 96-well plate seeded with either HEK-293 or L929 cells at 90% confluency. Cells were then incubated for 24 hr at 37 °C with 5.5% carbon dioxide. The initial and final cell necrosis after 24 hr were visualized using a fluorescent microscopy (Olympus CKX53, Olympus Life Science, Waltham, Massachusetts, US) and camera (Olympus EP50, Olympus Life Science, Waltham, Massachusetts, US). All samples were prepared in duplicate, with a control consisting of cells in growth media with PI, but without surfactants.

In the second control study, the effect of the PI dye on virus infectivity and viability was assessed. The purified viruses were mixed in 2 uM PI dye-infused virus media solution and then introduced to cell culture for one hr in 5.5% carbon dioxide in a 37 °C incubator. Then the virus growth media containing the dye solution was added and the samples were incubated for 24 hr. The control for this study included omitting the PI dye in the first one hr exposure of the viruses to cell culture, with the dye introduced to the sample after 24 hr of incubation. The assessment of cell death due to virus infection was assessed by fluorescent microscopy.

Finally, the interaction between viruses and different concentrations of surfactants from 0 mg/L to 12 mg/L with 2 mg/L increments was investigated. These surfactant concentrations were prepared in virus growth media-PI solution. 100 ul of surfactant-PI in virus media then mixed with 50 ul of purified viruses at a concentration of 10^7^ ADV or 10^5^ MHV TCID _50_/ml for 30 min at room temperature. The purified virus to surfactants volume ratio was set at 1:2 in 150 ul final volume. Then, 40 ul of virus-surfactants-PI solution was transferred to cell samples in a 96-well plate and incubated for 1 hour in 5.5% carbon dioxide 37 °C in an incubator to assist virus infectivity. Finally, 120 ul of virus media-PI solutions were added to each well to assist virus growth for 18–24 hours at 5.5% carbon dioxide 37 °C in an incubator. We assumed that the virus media-PI solution diluted the existing surfactant in well which decreased the effects of surfactants on cells. A 7-fold dilution series was used to assess the MIC for final surfactant concentration from 0 to 12 mg/l samples. All experiments used 96-well plates and were conducted in duplicate. The control consists of viruses with PI without surfactants (0 mg/L) and cell growth in regular cell media. After 24 hr of incubation, fluorescent microscopy (Olympus CKX53, Olympus Life Science, Waltham, Massachusetts, US) and camera (Olympus EP50, Olympus Life Science, Waltham, Massachusetts, US) were used to determine MIC in cell culture.

To validate the MIC dilution series results and evaluate if the surfactant exposure resulted in virus envelop and capsid rupture and nucleic acid release, a series of experiments were designed utilizing quantitative polymerase chain reaction (qPCR) to quantify the virus gene copies before and after surfactants exposure. It was assumed that enzyme treatment with RNase or DNase would degrade all free DNA or RNA released from the viruses if the surfactants disrupted the envelopes and capsids. Studies were undertaken to confirm that surfactants did not impact on the activity of DNase and RNase. The MIC assessment was conducted by mixing 150 ul of surfactants solution at various concentrations (0, 1, 2, 4, 6, 8, 16, 40 and 100 mg/L) with 100 ul of purified viruses. The mixture was left at room temperature for 60 min before treating with 5 ul of either DNase or RNase (Thermo Scientific, Waltham, Massachusetts, US). The sample was then incubated in a water bath for 15 mins at 37 °C. Upon removing samples from the water bath, virus DNA/RNA extraction was conducted following a previously published manual DNA/RNA extraction method (Griffiths et al. 2000). The experimental controls included the addition of 1X phosphate buffer solution in place of surfactants.

### Virus quantification via quantitative polymerase chain reaction (qPCR) methods

All oligonucleotides used as positive controls, primers, and probes were purchased from Integrated DNA Technologies (IDT-DNA, Coralville, Iowa, USA). Both RT-qPCR and qPCR assay were conducted using QuantStudio 3 (Thermo-Fisher Scientific, Waltham, Massachusetts, US). All qPCR and RT-qPCR runs included the corresponding positive control (oligonucleotides) and negative control (molecular grade water).

For the RT-qPCR assay, the MHV positive control was 5’ GGAACTTCTCGTTGGGCATTATACTTTTTTACATGCTACGGCTCGTGTAACCGAACTGTTTTTTTATGTTGTGAAAATGATAATCTTGTGGT -3’. The MHV RT-qPCR analysis was performed in duplicate 25 ul reaction mixtures which contain 20 ul of master mix (TaqPath^TM^ 1 step RT-qPCR MM, CG, Thermo-Fisher Scientific, Waltham, Massachusetts, US) and 5 ul MHV RNA samples. The MHV primer and probe sequences were used as previously reported (Li et al. 2022). The thermocycling conditions for MHV RT-qPCR were 50 °C for 15 mins, 95 °C for 2 mins, and 40 cycles of 95 °C for 10 s followed by 60 °C for 30 s. A six-fold dilution series of the positive control oligonucleotide was used to generate an MHV standard curve with a reaction efficiency of 89.1% and an R^2^ of 0.9936.

The ADV positive control was (5’-CACTCATATTTCTTACATGCCCACTATTTTTTTAGGAAGGTAACTCACGAGAACTAATGGGCCATTTTTCAATCTATGCCCAACAGGCC-3’). The ADV qPCR analysis was performed in duplicate 25 ul reaction mixtures which contains 20 ul master mix (TaqMan^TM^ Fast Advanced Master Mix, Thermo-Fisher Scientific, Waltham, Massachusetts, US) and 5 ul of ADV DNA samples. The ADV primer and probe sequences were previously reported (Ebner et al. 2006). The thermocycler conditions were 95 °C for 5 mins, and 40 cycles of 95 °C for 15 °C followed by 60 °C for 30 s. A six-fold dilution series of the positive control oligonucleotide was used to generate an ADV standard curve with a reaction efficiency of 90.4% and an R^2^ of 0.9998.

### Virus surface proteins and ionic surfactants via molecular dynamic modeling

To investigate the dynamics of the MHV Spike Glycoprotein S and its interactions with a lipid bilayer and surfactants, molecular dynamics (MD) simulations were performed on two carefully designed systems. The spike protein from MHV-A59 (the RCSB PDB ID: 1WDF) (Xu et al. 2004) was selected due to its high-resolution structure, minimal missing residues, and its relevance as a representative surface protein of the virus. This choice ensures structural integrity and reliability in the simulation outcomes, enabling mechanistic comparisons with experimental observations. While the simulations do not model the entire virus, they provide atomistic insight into how surfactants perturb key viral components embedded in a realistic membrane context. The MHV Spike Glycoprotein S was embedded in a symmetrical bilayer composed of the lipids 188 POPC (1-palmitoyl-2-oleoyl-sn-glycero-3-phosphocholine), 60 CHL (cholesterol), 44 POPI (1-palmitoyl-2-oleoyl-sn-glycero-3-phosphoinositol), 80 POPE (1-palmitoyl-2-oleoyl-sn-glycero-3-phosphoethanolamine), and 28 POPS (1-palmitoyl-2-oleoyl-sn-glycero-3-phospho-L-serine), solvated with ∼48 200 TIP3P water molecules (Jorgensen et al. 1983), and neutralized with 353 ions to achieve an ionic strength of 150 mM NaCl. Additionally, ten surfactant molecules were randomly introduced to investigate their binding modes and their potential to modulate protein-membrane interactions. The system contained approximately 199 000 atoms within a periodic simulation box measuring 10.65036 × 10.65036 × 17.01 209 nm after energy minimization. The CHARMM36 m force field was employed for all simulations, ensuring an accurate representation of protein-surfactant, protein-lipid, and lipid-surfactant interactions (Huang and MacKerell Jr 2013, Huang et al. 2017). The SDS molecular parameters were derived using the CHARMM General Force Field (CGenFF, version 2.5.1) (Vanommeslaeghe et al. 2010, Vanommeslaeghe and MacKerell Jr 2012). Input preparation for these molecules was performed using *psf2itp.py*, a utility designed for GROMACS (Van Der Spoel et al. 2005) input generation with the CHARMM36 force field (Vanommeslaeghe and MacKerell Jr 2012). The parameterization ensured the compatibility and accuracy of the surfactant interaction models with the protein and lipid components of the system. Protein topology and coordinates were prepared using the CHARMM-GUI Membrane Builder (Jo et al. 2009) was employed to insert the protein into the system, positioning it in close proximity to the membrane surface to allow for immediate interaction. The solvated and neutralized system, including the surfactants, was carefully checked for consistency before proceeding to minimization and equilibration. All simulations were run with the GROMACS 2024.2 version (Abraham et al. 2015).

Energy minimization was performed using the steepest descent algorithm until the maximum force converged below 1000 kJ/mol/nm, removing steric clashes and reducing high-energy contacts. Following minimization, the system underwent a six-step equilibration protocol to gradually relax restraints and stabilize the environment. The timestep was set to 1 fs for the first three steps and increased to 2 fs for the final three. Position restraints on the protein backbone, sidechains, and lipids were progressively reduced across the six steps (from 4000 → 2000 → 1000 → 500 → 200 → 50 kJ/mol/nm² for the backbone; 2000 → 1000 → 500 → 200 → 50 → 0 kJ/mol/nm² for sidechains; and 1000 → 400 → 400 → 200 → 40 → 0 kJ/mol/nm² for lipids). Dihedral restraints were similarly relaxed (1000 → 400 → 200 → 200 → 100 → 0 kJ/mol/nm²) to allow for sidechain and secondary structure relaxation. Temperature coupling was applied using the velocity-rescale thermostat (Bussi et al. 2007), maintaining a target temperature of 303.15 K for three distinct groups (protein, lipids, and solvent), while pressure coupling was introduced in step 6.4 using a semi-isotropic compressibility scheme and a reference pressure of 1 bar via the C-rescale algorithm (Bernetti and Bussi 2020), ensuring proper equilibration of system dimensions and density. Non-bonded interactions were treated with the Verlet cutoff scheme (Verlet 1967), applying a force-switch modifier for van der Waals interactions starting at 1.0 nm and extending to 1.2 nm. Long-range electrostatics were calculated using the Particle Mesh Ewald (PME) method (Essmann et al. 1995), with a cutoff of 1.2 nm for short-range interactions. The production simulations were run for 1 microsecond to provide detailed insights into the structural dynamics of the MHV Spike Glycoprotein S, its interaction with surfactants, and their potential to modulate protein-lipid-surfactant contacts. The analysis included identifying surfactant binding sites, evaluating the stability of protein-surfactant complexes, and determining whether surfactants influence the penetration depth or orientation of the protein within the lipid bilayer. Trajectories were saved every 5000 steps (10 ps), enabling high-resolution monitoring of system behavior throughout equilibration and production.

System visualizations and figure generation were carried out using VMD 1.9.3 and ChimeraX 1.8 (Humphrey et al. 1996, Goddard et al. 2018, Meng et al. 2023), which facilitated the interpretation of structural features, protein-lipid interactions, and surfactant binding sites. In-house Python scripts and GROMACS tools were employed for trajectory analysis, including calculations of binding site probabilities of the surfactants interacting with the protein (cutoff = 4.0 Å), and identifying individual residues with high binding probability, and to compute contact frequency distributions as a function of interaction distance. These scripts generated histograms of both raw and normalized contact frequencies. Additionally, the APBS server (Jurrus et al. 2018) was used to obtain the electrostatic potential maps for the protein in each simulation, utilizing the most populated cluster of the final 100 ns of the trajectory for these calculations. Specific attention was given to the interaction energies between the surfactants and the protein, as well as to changes in membrane properties induced by the surfactants. All input files, parameter settings, and Python scripts are available upon request to ensure reproducibility.

### Statistical methods

Two-way ANOVA was used to compare the dependent variable of virus hydrated radius as a function of the two independent variables of pH (3 to 9) and surfactant concentration (0, 5 and 10 mg/L). When the dataset contained observations at all pH and surfactant levels (i.e. excluding only ADV with 10 mg/L BAC at pH 3 to 7) interactions among pH and the surfactant level were also assessed. When the independent variables were a source of variation in the ANOVA, a Holm-Sidak pairwise comparison was used to determine if any group means were significantly different for each factor, with an overall significance level of 0.05.

## Results

### Virus harvesting and purification

In preparation for the purification process, virus stocking solutions were thawed at room temperature. Each freeze and thaw cycle of the virus stocking solution was found to decrease the virus count between 10^2^ to 10^3^ TCID _50_/ml. Infectivity of the MHV virus was observed to decrease when the MHV virus was incubated beyond 24 hr, with no infectious MHV viruses remaining after a 48 hr incubation period. In contrast, ADV demonstrated sustained infectivity for up to 48 hr in the incubator. The sucrose cushion purification method increased the MHV viral titer from 10^2^ to 10^5^ TCID _50_/ml. Similarly, the ADV viral titer increased from 10^7^ to 10^10^ TCID _50_/ml. Purified viruses could be stored in virus medium at 4 °C for an extended period without affecting their viability.

### Virus hydrated diameter with surfactants

The DLS-PALS zeta potential analyzer was used to estimate the hydrodynamic radius of the viruses after exposure to surfactants at different pH. The results indicate that surfactants influence the hydrated diameter of enveloped and non-enveloped viruses differently. Herein, at all pH tested, the MHV hydrated diameter in buffer solution was 150 ± 10 nm (average ± standard deviation), while the ADV hydrated diameter was 142 ± 8 nm (Table S1, supplemental information). There was no significant difference in the hydrated diameter of ADV at different pH in BAC (*P* = 0.30, n = 138) or SDS (*P* = 0.338, n = 130) buffer solution. Similarly, for MHV when pH differed there was no significant difference observed in the hydrated diameter when BAC (*P* = 0.68, n = 120) or SDS (*P* = 0.94, n = 212) were also present. However, there was a significant difference in ADV diameter in the presence of SDS (*P* = 0.03, n = 212) and BAC (*P* < 0.001, n = 138). At 5 and 10 mg/L BAC, the diameter of ADV significantly decreased from 142 nm to 109 ± 21 nm (*P* < 0.001, Holm-Sidak pairwise comparison) and 115 ± 21 nm (*P* = 0.002, Holm-Sidak), respectively. For MHV, while SDS and BAC decreased the mean hydrated radius (Table S1), only SDS significantly changed the hydrated radius of MHV (*P* = 0.03, n = 212) and only for the 5 mg/L SDS treatment (*P* = 0.034, Holm-Sidak). Specifically, the hydrated radius of MHV decreased in the presence of 5 mg/L SDS to 136 ± 28 nm (Table S1) from 150 ± 10 nm (no surfactant present). While any surfactant was shown to decrease the hydrated diameter of ADV, there was no significant difference in the ADV hydrated diameter when BAC was either 5 and 10 mg/L (*P* = 0.18, Holm-Sidak), nor when SDS was either 5 or 10 mg/L (*P* = 0.63, Holm-Sidak), suggesting any surfactant below the MIC was likely to reduce the hydrated diameter of ADV.

### Virus isoelectric point with surfactants

**Figure 3.**
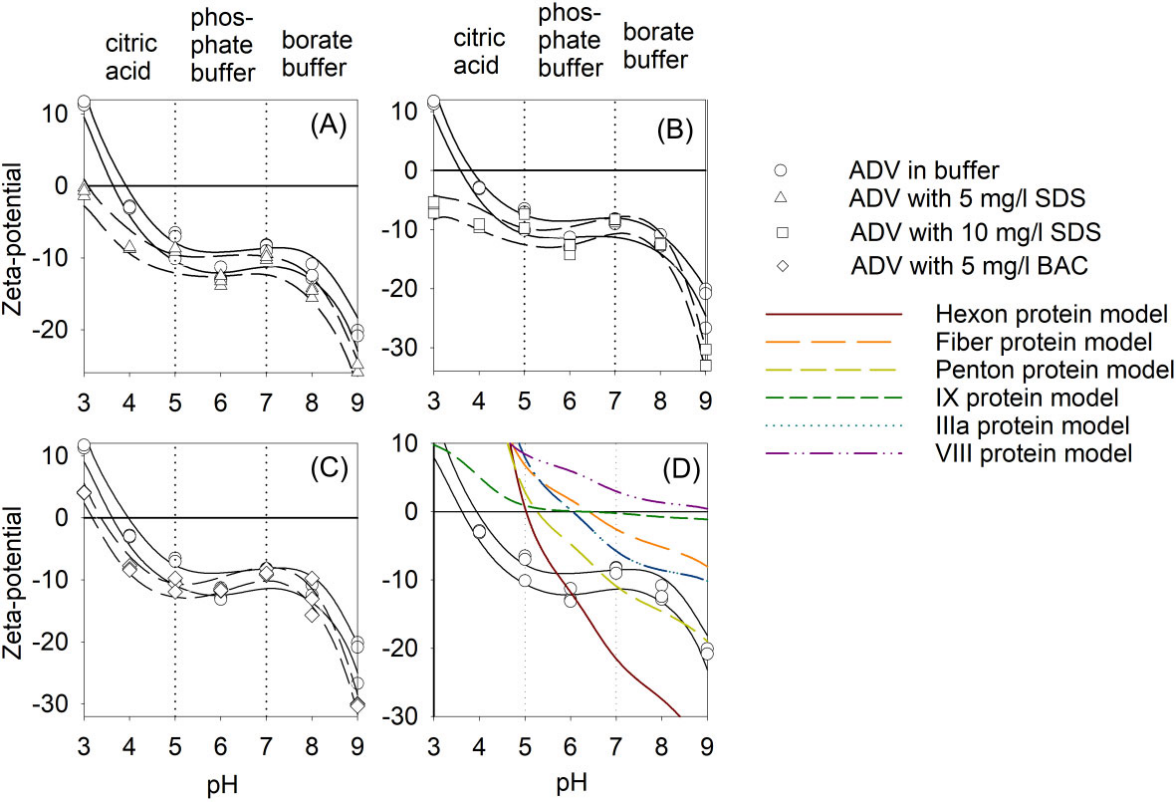
Observed ADV zeta-potential at various pH with and without surfactants (symbols), 95% confidence intervals on zeta-potential (black lines) and modeled zeta-potentials of ADV surface proteins as a function of pH. All solutions have an ionic strength of 0.1 M. The IEP was assumed to be the midpoint of the 95% confidence interval lines that crossed the zero zeta potential line.

**Figure 4.**
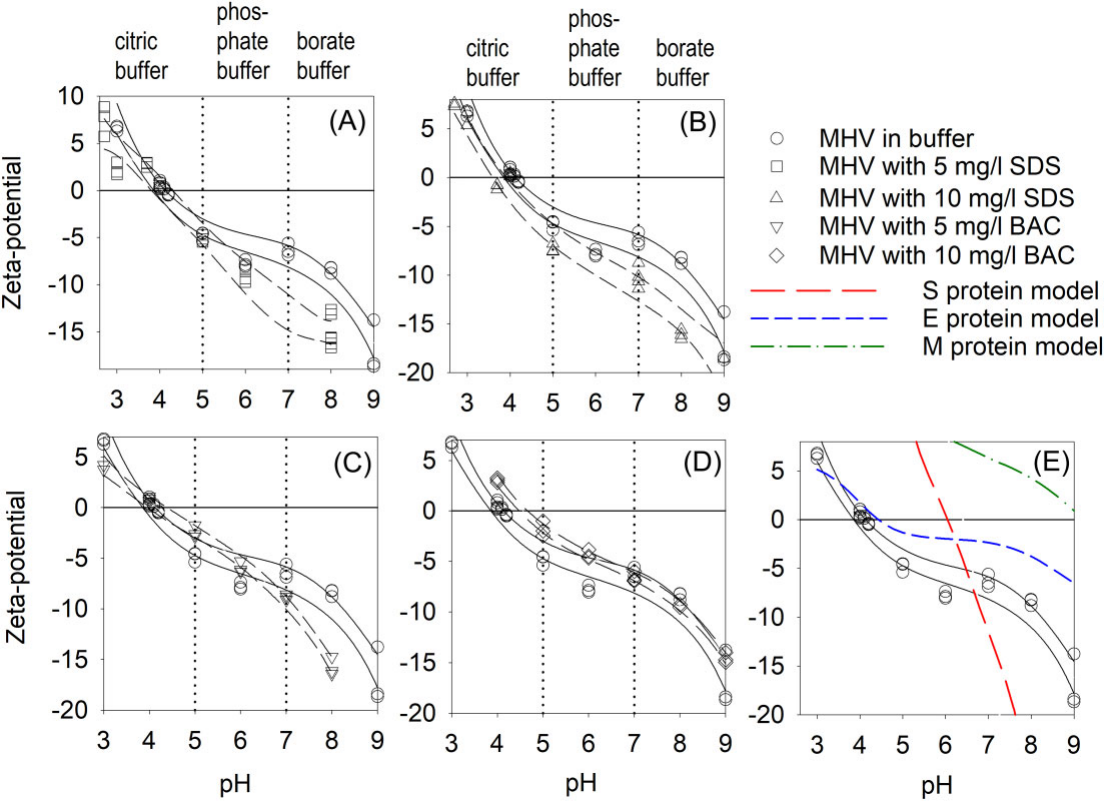
Observed MHV zeta-potential at various pH with and without surfactants (symbols), 95% confidence intervals on zeta-potential (black lines) and modeled zeta-potentials of MHV surface proteins as a function of pH. All solutions have an ionic strength of 0.1 M. The IEP was assumed to be the midpoint of the 95% confidence interval lines that crossed the zero zeta potential line.

The virus zeta potential at different pH and surfactant concentrations are shown in Figs. 3 and 4. Using the midpoint of the 95% confidence intervals of the observed data, the IEP was determined to be 3.8 for ADV without any exposure to surfactants, while exposure to 5 mg/L BAC reduced the IEP for ADV to 3.3 (Fig. 3a). While not observed experimentally herein, the IEP for ADV exposed to 5 mg/L SDS was less than pH 3 and using the 95% confidence interval curves estimated to be pH 2.9 (Fig. 3a). The IEP for ADV exposed to 10 mg/L SDS (Fig. 3b) or BAC (only pH 7, 8 and 9 studied) was not estimable in this study as the zeta potential never approached neutrality. Therefore, the results herein suggest that addition of surfactants at concentrations below the MIC will lower the enveloped virus ADV IEP by 0.5 to 0.8 pH. Further, these experimentally observed IEP are lower than those predicted by bioinformatic modeling of the surface proteins alone as shown in Table 1 for ADV. Ranges of predicted IEPs for ADV surface proteins ranged from 5.1 to 6.3 (Table 1 and Fig. 3D).

**Table 1.** Estimated ADV IEP using EMBOSS and Expasy.

ADV PROTEIN NAme ^a^	ADV protein ID (Uniprot)	EMBOSS IEP	Expasy IEP
Hexon	P04133 CAPSH_ADE05	5.1	5.2
Fiber	P11818|SPIKE_ADE05	6.4	6.0
Penton	P12538 · CAPSP_ADE05	5.3	5.4
IX	P03281|CAP9_ADE05	6.3	6.1
IIIa: Pre-hexon-linking protein IIIa	Q6VGV1_ADE05	6.0	5.8
VIII: Pre-hexon-linking protein VIII	Q6VGU2_ADE05	9.2	9.0

a . proteins are shown in Fig. 2.

The IEP for MHV was also impacted with the addition of surfactants but primarily at 10 mg/L SDS levels. Based on the mid-points of the 95% confidence intervals of the observed data, the IEP for MHV without exposure to surfactants was 3.99 pH (Fig. 4a). The IEP of MHV generally increased when SDS was added and was estimated to be pH 4.16 for 10 mg/L SDS. The IEP of MHV when exposed to BAC varied, decreasing to pH 3.74 when exposed to 5 mg/L BAC and increasing to 4.56 when exposed to 10 mg/L BAC. In contrast to ADV, the use of bioinformatic modeling was better at estimating the IEP for MHV, especially for the envelop small membrane protein (Table 2) and for modeling the zeta potential at different pH (Fig. 4E). Therefore, the results herein suggest that when exposed to surfactants SDS, the non-enveloped MHV IEP will increase between 0.2 to 0.6 pH.

**Table 2.** Estimated MHV IEP using EMBOSS and Expasy

MHV protein name ^a^	MHV protein ID (Uniprot)	EMBOSS IEP	Expasy IEP
Membrane protein, M protein, E1 glycoprotein, Matrix glycoprotein, Membrane glycoprotein	P03415 VME1_CVMA5	9.2	9.1
Envelope small membrane protein (E)	P0C2R0 VEMP_CVMA5	4.4	4.4
Spike Glycoprotein S	P11224 · SPIKE_CVMA5	6.1	5.8

a. proteins are shown in Fig. 2.

For most environmental applications, such as during water or wastewater treatment pH near neutral will dominate. Small changes in the zeta potential of nanoparticles and viruses can influence the efficacy of treatment technologies and virus-surface interactions. Therefore, we also determined the zeta potentials at pH 7 for ADV and MHV when exposed to different surfactant concentrations. Again, using the polynomial equation for the 95% confidence intervals to determine the zeta potential at pH 7, it was found that the ADV IEP did not differ much with and without surfactants and included -9.95 mV (no surfactants), -11.08 mV (5 mg/L SDS), -9.29 mV (10 mg/L SDS), and -9.09 mV (5 mg/L BAC). However, the zeta potential for MHV was generally lower when surfactants were present, dropping from -7.00 mV at pH 7 with no surfactants, to -11.4 and -12.9 mV when 5 and 10 mg/L BAC was present respectively. The zeta potential of MHV at pH 7 with 5 mg/L SDS was -6.5 mV and for 10 mg/L SDS was -9.4 mV. These results suggest that surfactants present at low concentrations change the surface charges on viruses and thus may impact virus surface interactions in water systems.

### Minimum inhibitory concentration

Both cell culture and quantitative PCR (qPCR) methods were employed to determine the minimum inhibitory concentrations (MICs) of the surfactants that inhibit the infectivity of viruses. To our knowledge, this study is the first to report the MIC of these surfactants for MHV and ADV under defined conditions. Prior to assessing the MIC, preliminary tests showed that the PI dye did not impact on the infectivity of the viruses over 24 hr of direct incubation. Further, after 24 hr of exposure to surfactants, more than 90% of L929 cells survived at BAC concentrations of ≤ 4 mg/L and SDS concentrations of ≤ 12 mg/L. Similarly, 60–70% of HEK-293 cells remained viable at BAC and SDS concentrations of ≤ 4 mg/L. Consequently, the established MIC for L929 cells were 4 mg/L for BAC and 12 mg/L for SDS, whereas for HEK-293 cells, both surfactants had MIC of 4 mg/L. Then, when exposing viruses to SDS or BAC and then incubating the viruses with the appropriate cell lines, the MICs for all surfactant and virus combinations were determined to be 1 mg/L, over a 60-minute exposure at room temperature followed by a 24 hr incubation period. This result was validated via qPCR, which confirmed by quantifying the residual viral nucleic acids and demonstrated that the surfactants did not inhibit the activity of DNase/RNase enzymes or the qPCR amplification itself.

### Molecular dynamic modeling

**Figure 5.**
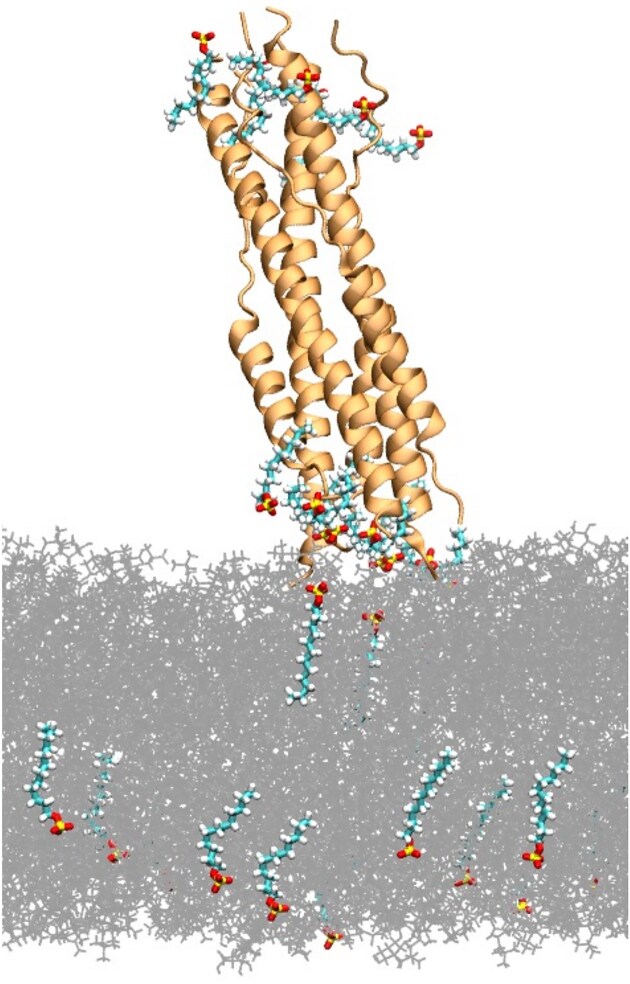
MHV spike protein with SDS molecules interacting with the lipid bilayer. The protein is shown in orange cartoons, the lipids in gray lines, and the SDS molecules with sticks. Carbons in green, hydrogens in white, oxygen in red, and sulfur in yellow.

In order to understand the atomistic details of how surfactants interact with the MHV Spike Glycoprotein S and influence its behavior, MD simulations were performed. These simulations aim to provide an in-depth perspective on the binding sites, structural dynamics, and electrostatic potential alterations induced by surfactants, complementing the experimental observations. To facilitate the observation of multiple interaction events within a feasible simulation timescale, surfactants were introduced at concentrations higher (17.3 mg/L for SDS and 27.4 mg/L for BAC) than those used experimentally. This design choice enabled the identification of distinct binding regions and interaction patterns without compromising the atomistic resolution of the system. Notably, the interaction between the protein and the lipid bilayer was significantly reduced in the presence of surfactants. Surfactant molecules predominantly accumulated at the top and bottom extremes of the protein, suggesting specific regions of higher affinity. Both SDS and BAC demonstrated the ability to easily move through the lipid bilayer, indicating a high degree of membrane permeability. Multiple interactions were observed between the surfactants and the aliphatic residues on the protein surface. SDS exhibited interactions with both aliphatic and charged residues, highlighting its potential for electrostatic interactions in Fig. 5.

**Figure 6.**
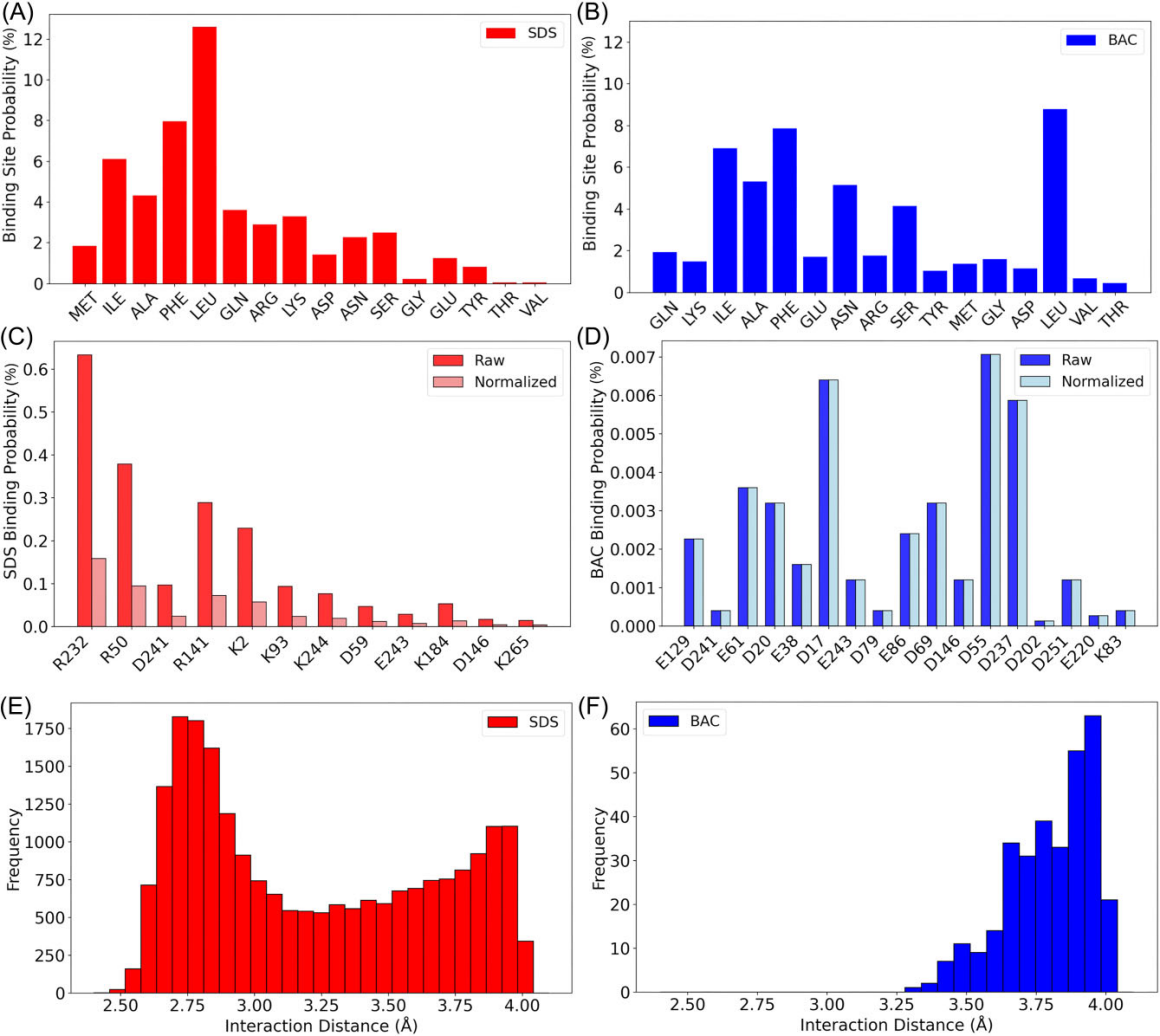
Binding probabilities for each charged residue are shown for SDS (plot A) and BAC (plot B). These represent the fraction of simulation frames in which a residue is in contact with any surfactant atom. A focused view of key charged residues (plots C and D) that exhibit high binding probabilities. Histograms (plots E and F) of the raw contact frequencies, plotted as a function of interaction distance.

To further elucidate the specific interactions between surfactants and the protein, binding site probabilities were calculated. For SDS, significant interactions were identified with residues such as M, I, A, F, L, Q, R, K, D, N, S, G, E, Y, T, and V. Among these, SDS showed a higher probability of interacting with residues like I (6.10%), F (7.95%), and L (12.59%), indicating a preference for hydrophobic interactions. Charged residues such as R, K, D, and E also displayed notable interaction probabilities, emphasizing the role of electrostatic forces. Specific interactions of SDS with positive residues (R, K) and negative residues (D, E) are shown in Fig. 6a. Similarly, BAC interactions were analyzed, revealing significant binding probabilities with residues such as Q, K, I, A, F, E, N, R, S, Y, M, G, D, L, V, and T. BAC demonstrated strong interactions with residues like I (6.90%), F (7.84%), and L (8.76%), similar to SDS. The interaction pattern with charged residues was slightly different, with R, K, D, and E showing notable probabilities. Specific interactions of BAC with charged residues were also highlighted, showing a balanced interaction pattern between positive (R, K) and negative (E, D) residues (Fig. 6b). Both surfactants exhibited substantial interactions with neutral amphiphilic groups, with SDS interacting slightly more with positive residues compared to BAC.

High-probability-specific interactions were further analyzed by determining the binding site probabilities for each charged residue. In our analysis, the raw binding probability is defined as the fraction of simulation frames in which a given residue is in contact (within a 4.0 Å cutoff) with any surfactant atom. To account for the multivalency of surfactants, we distinguish between raw and normalized probabilities. SDS possesses four sulfate oxygens capable of forming electrostatic contacts, whereas BAC contains only a single charged nitrogen. Therefore, raw binding probabilities for SDS were divided by four to yield an effective per-site probability, allowing a more fair comparison across surfactants. For SDS (Fig. 6c), several key basic residues exhibited notably strong interactions. For example, residue R232 had a raw binding probability of approximately 63.3%, which, when divided by 4, yields an adjusted probability of about 15.8%. Similarly, R50 and R141 showed raw probabilities of roughly 37.9% and 28.9%, corresponding to adjusted values of approximately 9.5% and 7.2%, respectively. In addition, select lysine residues also demonstrated significant binding: K2 exhibited a raw value of around 22.9% (adjusted to 5.7%), and K244 showed around 7.6% (adjusted to 1.9%). Among the acidic residues, D241 was contacted in roughly 9.7% of frames (adjusted to 2.4%) and D59 in about 4.7% (adjusted to 1.2%). These results indicate that SDS's multivalent interactions—primarily with basic residues, but also with selected acidic residues—are substantially stronger and more frequent. In contrast, BAC (Fig. 6d) consistently shows much weaker associations across all charged residues. Its highest binding probabilities remain uniformly below approximately 1%—for instance, D17 is contacted in about 0.64% of frames and D55 in roughly 0.71%. Even without any adjustment (given BAC's single charged site), these values underscore a markedly lower binding capacity compared to SDS.

In addition, the contact frequency distributions were analyzed as a function of interaction distance. The histogram of raw contact counts reveals that SDS interactions (Fig. 6e) occur very frequently over a broad distance range, from approximately 2.5 Å to 4.0 Å, with a peak frequency reaching up to 1750 counts near 2.75 Å. Conversely, BAC interactions (Fig. 6f) are considerably sparser, with frequency counts not exceeding about 65 and contacts occurring within a narrower distance window, from roughly 3.3 Å to 4.0 Å, with a peak near 3.90 Å. This stark contrast in both the magnitude and the distance profile of contacts further illustrates the much stronger and closer associations of SDS with the spike protein compared to the more diffuse and weaker interactions observed for BAC.

**Figure 7.**
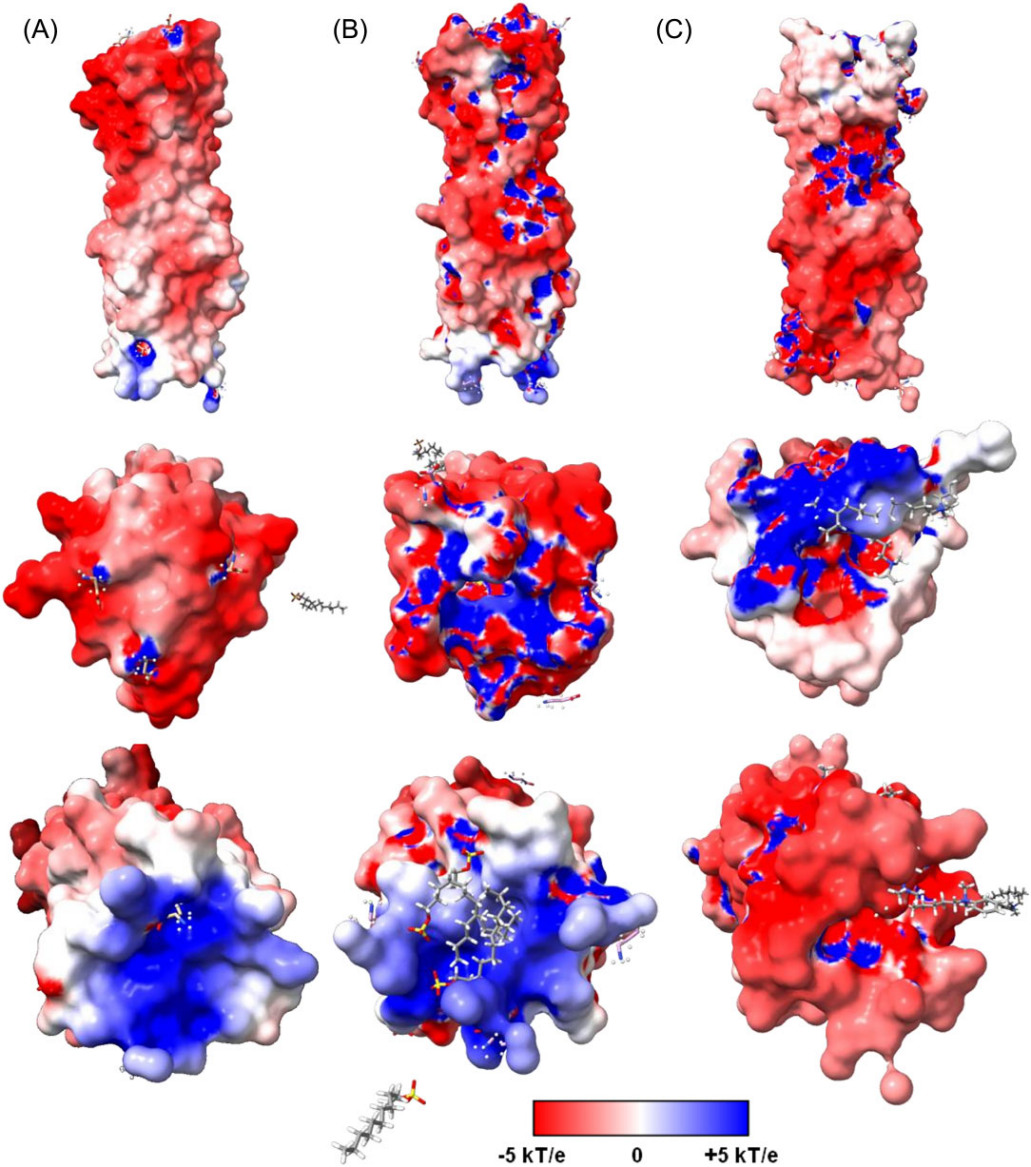
Electrostatic potential maps of the MHV Spike Glycoprotein S were obtained using the APBS server, shown in side, top, and bottom orientations for the unbound protein, with SDS, and with BAC. Red denotes negatively charged regions and blue denotes positively charged regions. Relative to the unbound state, SDS produces a broader increase in positive potential across top, bottom, and lateral surfaces, whereas BAC induces localized increases at the top.

To complement these findings, APBS analysis was conducted to obtain electrostatic potential maps for the protein in each simulation condition. The electrostatic potential maps revealed several critical observations. In regions where surfactants interact, particularly at the top and bottom extremes of the protein, a visible neutralization of charge was observed. This shift from red to white or blue to pale indicates the impact of surfactant binding on the protein's electrostatic potential. Notably, more SDS molecules were observed interacting between the protein and the membrane, suggesting a more substantial effect on the protein's electrostatic environment. This accumulation of SDS effectively neutralizes and “blocks” positive charge regions on the protein, potentially destabilizing the virus by disrupting its electrostatic interactions with the membrane. Quantitative analysis of the electrostatic potential maps revealed that the total electrostatic energy of the protein with SDS was significantly lower (1.96e^5^ kJ/mol) compared to the protein with BAC (2.20e^5^ kJ/mol) and the protein alone (2.39e^5^ kJ/mol). These values further highlight the stronger impact of SDS on altering the protein's electrostatic properties (Fig. 7). The APBS analysis supports the experimental findings, particularly highlighting the better efficacy of SDS in altering the virus's electrostatic properties and contributing to viral destabilization.

These results from the detailed MD simulations underscore the importance of electrostatic and hydrophobic interactions in modulating protein behavior and stability, contributing to a deeper understanding of how surfactants influence viral properties and informing understanding of virus-surface interactions in water, wastewater and environmental systems.

## Discussion

Herein, we used dynamic light scattering (DLS) to examine the virus-hydrated diameter by measuring the intensity of scattered light as a function of time due to the particle's Brownian motion (Stetefeld et al. 2016). Previous literature reported that MHV has an absolute diameter ranging from 80-120 nm by cryo-electron tomography, while ADV has a diameter of 90 nm (Bárcena et al. 2009, Saha and Parks 2020, Heffron and Mayer 2021, Takuissu et al. 2024). However, our measurement reveals an increase in hydrated diameter in a buffer solution of MHV 150 ± 10 nm (average ± standard deviation), while the ADV hydrated diameter was 142 ± 8 nm (Table S1, supplemental information). This is expected, as the hydrated diameter of the viruses is typically larger than their absolute diameters because colloidal particles form double electric layers in solution (Maguire et al. 2018). For both viruses, different pH solutions did not significantly affect the virus-hydrated diameters. These observed results can likely be attributed to the same buffer solution ionic strength which is 0.1 M, which potentially hindered particle aggregation (Furukawa et al. 2009). As others have reported that the buffer concentration can influence the apparent hydrated diameter, (i.e. increasing NaCl concentrations decreased apparent hydrated diameter from 33 nm to 30 nm (Vodolazkaya et al. 2022)) we were careful in our studies to use one ionic strength solution for all experiments.

Our results show that addition of surfactants reduce virus-hydrated diameters (supplemental information). One possible explanation is that the Smoluchoski model, used to calculate all particle hydrated diameters, is generally accurate for large colloidal particles (with radii above 10 nm) in high salt solutions (Polaczyk et al. 2020). However, this model may not be accurate for all virus particles, leading to errors in calculating both particle size and zeta potential (Polaczyk et al. 2020). Additionally, particle size calculated using DLS is based on the Stokes-Einstein equation, which is proportional to the sixth power of the particle diameter and solvent viscosity (Maguire et al. 2018). In this case, the DLS analysis will be biased towards the larger particle sizes and will indicate two peaks in the analysis (Maguire et al. 2018). Solvent viscosity, which is related to the particle Brownian motion, also plays a role. In this case, increased surfactant concentration in a buffer may affect the particle size through van der Waals interaction and viruses- surfactant binding in solution (Maguire et al. 2018). Finally, these results could suggest that the low concentration surfactants are thinning or dissolving envelop bilayers (Becker et al. 1975, Negi et al. 2024), stripping the membrane of surface spike proteins (Simon et al. 2021), or altering the virus protein configuration to form more compact molten-globule states before unfolding at higher surfactant concentrations (Moosavi-Movahedi et al. 2003, Xu and Keiderling 2004). Decreases in hydrodynamic diameter of MS2 particles with the addition of SDS and Triton X-100 was also observed by others (Vodolazkaya et al. 2023) although the decrease in diameters decreased by only 2 to 4 nm (i.e. 35 nm to 33 nm with SDS and 34 to 30 nm with Triton X-100).

The phase analysis light scattering (PALS) method measures particle mobility in an electric field (Thomas et al. 2002). The distance of a particle moved per electrical field based on time will be used to calculate zeta potential. The isoelectric point (IEP) is reached when the particle's net charge is neutral, a measurement based on the two layers of liquid surrounding the particle (Bhatia and Dahiya 2015). Our finding for the ADV IEP using PALS is 3.8. Comparative analysis against the literature findings revealed the ADV IEP was at a pH of 2.6 using the method isoelectric focusing on dense aqueous solution (IEF-DA) (Michen and Graule 2010). Differences between these two experimentally determined IEPs may be attributed to multiple factors, including the chosen methods, buffer types, buffer ionic strength, and the types of charged ions present in the solution (Michen and Graule 2010, Samandoulgou et al. 2015). The IEF-DA method requires a viral target fluorescent tag which requires pure, concentrated virus solution (Areo et al. 2021). Conversely, zeta-potential measurement is more related to the virus-surrounded solution. For example, both virus solubility in solution and adsorption interactions between the virus surface and inorganic solutes in the buffer will alter virus IEP (Lützenkirchen et al. 2018, Areo et al. 2021). There is no existing IEP estimation for MHV viruses due to enveloped virus glycosylation and lipid bilayers making the surface characterizations more difficult to predict (Areo et al. 2021). This can be also used to explain at pH 7 ADV IEP did not differ much with and without surfactants Conversely, MHV zeta-potential dropped from -7.00 mV at pH 7 with no surfactants, to -11.4 and -12.9 mV when 5 and 10 mg/L BAC was present respectively. The zeta potential of MHV at pH 7 with 5 mg/L SDS was -6.5 mV and for 10 mg/L SDS was -9.4 mV.

Our experimental IEP results for both MHV and ADV were lower than the modeling results that were based on virus surface amino acid structures alone. The discrepancy may arise from the modeling tool's assumption of either free-floating proteins or completely denatured polypeptides for calculating protein IEP, while the virus existed as a complete particle with charged groups residing internally. Since these groups were shielded, they contributed negligibly to the zeta potential. This could lead to a deviation between the model and measurement. The post-translational modification especially for enveloped viruses was not considered in the Expasy modeling tool (Areo et al. 2021). The modeled MHV and ADV surface proteins will exhibit maximum steepness at the pH values corresponding to their acid dissociation constant at the logarithmic scale (pKa) of the distinct amino acid side chains (Zhou and Pang 2018, Tokmakov et al. 2021). The contribution of each amino acid to the curve depended on its abundance in the modeled proteins. The modeled IEP curve indicated that single protein folding and unfolding significantly impacted the IEP curve. However, both modeling tools ignored the solution ionic strength effects when determining the IEP, as the results obtained with and without factoring in ionic strength yield little to no deviation. The E protein of MHV modeled in Fig. 3E, exhibits trends like those observed in the experimental virus IEP. This suggests that the E protein of MHV may be the principal surface protein influenced by SDS and BAC. Further, the lipids in the MD modeling should significant interaction with the SDS molecule (Fig. 5 and 7). Similarly, ADV possesses three major surface proteins. Data presented in Fig. 3D, suggests that surfactants may affect all three major surface proteins of ADV to influence the ADV IEP. EMBOSS and Expasy IEP modeling results differed from our experimental virus IEP results, suggesting that these models were insufficient to capture complex surfactant effects on virus surface properties.

The computational results provide a detailed atomistic perspective on the interactions between surfactants and the MHV Spike Glycoprotein S, complementing the experimental observations. These simulations elucidate how surfactants affect the protein's structure, behavior, and electrostatic properties. Our MD simulations demonstrated that the presence of surfactants significantly disrupts the interaction between the MHV Spike Glycoprotein S and the lipid bilayer. This finding aligns with the experimental observations of changes in the IEP and zeta potential of the virus upon exposure to surfactants. The simulations revealed that surfactant molecules predominantly accumulate at the top and bottom extremes of the protein, suggesting specific regions of higher affinity for surfactant binding. This accumulation likely contributes to the observed disruption of protein-membrane interactions. The multiple interactions observed between surfactants and aliphatic residues on the protein surface, especially those involving charged residues in the case of SDS, underscore the importance of both hydrophobic and electrostatic interactions in surfactant binding. Furthermore, both SDS and BAC were found to permeate the lipid bilayer easily, indicating high membrane permeability (Fig. 5). This behavior is crucial for understanding how surfactants interact with viral proteins in complex environments such as water and wastewater treatment systems.

Detailed binding site probability analysis provided further clarity on the specific residues involved in surfactant interactions. SDS (Fig. 6a) displayed significant interaction probabilities with a range of residues, including I, F, L, R, K, D, and E. The higher probability of SDS interacting with hydrophobic residues such as I and L underscores the role of hydrophobic forces, while the interactions with charged residues like R, K, D, and E indicate strong electrostatic attractions. In contrast, BAC interactions exhibited a slightly different pattern (Fig. 6b), with significant probabilities for residues like I, F, L, R, K, D, and E. The interactions of BAC were more evenly distributed among positively and negatively charged residues, as shown in Fig. 6B. This balanced interaction pattern suggests that BAC may have a more generalized effect on the protein surface, interacting with both hydrophobic and charged groups. The analysis of high-probability-specific interactions has revealed striking differences between the two surfactants (Fig. 6C and 6D). SDS, with its four sulfate oxygens, engages in multivalent interactions that yield raw binding probabilities as high as 63.3% for residues such as R232. Even after adjusting for multivalency (by dividing by 4), effective binding probabilities for key charged residues—R232, R50, and R141—remain in the range of 15.8%, 9.5%, and 7.2%, respectively. Additionally, considerable interactions were observed for lysine residues (for example, K2 and K244) and even for selected acidic residues (such as D241 and D59), suggesting that while electrostatic attraction with positively charged residues dominates, hydrophobic forces and local environmental effects may facilitate contacts with acidic sites as well. In contrast, BAC exhibits a uniform binding pattern where no residue achieves a binding probability above approximately 1%. This stark disparity is caused by the steric effect of the ring group in the BAC molecule and highlights the superior binding capacity of SDS due to its multivalent sulfate headgroup.

The contact frequency distributions further reinforce this conclusion (Fig. 6E and 6F). For SDS, the histogram of interaction distances shows a broad distribution, with contact counts peaking at around 1750 near 2.75 Å and extending from approximately 2.5 Å to 4.0 Å. These data indicate that not only does SDS form frequent contacts, but these contacts also occur at very close distances, which are critical for establishing strong electrostatic and hydrophobic interactions. In contrast, BAC interactions are markedly less frequent, with maximum counts registering only around 65; contacts occur within a much narrower distance range (approximately 3.3 Å to 4.0 Å, peaking near 3.90 Å). This difference in both the magnitude and spatial distribution of contacts underscores that SDS engages in much stronger and closer interactions with the protein than does BAC.

Electrostatic potential analysis, conducted using the APBS server, provided additional understanding of the effect of surfactants on the protein's electrostatic environment. The electrostatic potential maps revealed visible neutralization of charge in regions where surfactants interact, particularly at the top and bottom extremes of the protein. This neutralization is characterized by a shift from red to white or blue to pale, indicating the impact of surfactant binding on the protein's electrostatic potential. Notably, more SDS molecules were observed interacting between the protein and the membrane, suggesting a more substantial effect on the protein's electrostatic environment. This accumulation of SDS effectively neutralizes and “blocks” positive charge regions on the protein, potentially destabilizing the virus by disrupting its electrostatic interactions with the membrane. These findings are depicted in Fig. 7, which shows the electrostatic potential maps for the protein with SDS, without surfactants, and with BAC. Quantitative analysis of the electrostatic potential maps showed that the total electrostatic energy of the protein with SDS was significantly lower (1.96e^5^ kJ/mol) compared to the protein with BAC (2.20e^5^ kJ/mol) and the protein alone (2.39e^5^ kJ/mol). These values further highlight the stronger impact of SDS on altering the protein's electrostatic properties.

Overall, the computational results support and extend the experimental findings, providing a comprehensive understanding of how surfactants interact with the MHV Spike Glycoprotein S at an atomistic level. The integration of binding site probability data with contact frequency distributions reveals that SDS's multivalent sulfate group not only confers a considerably higher binding propensity for key charged residues (notably R232, R50, R141, K2, and D241), but also promotes more intimate contacts with the protein. In contrast, BAC's interactions are sparse, occur at longer distances, and are less likely to induce large perturbations in the protein's electrostatic landscape. The detailed MD simulations and electrostatic potential analyses highlight the importance of both hydrophobic and electrostatic interactions in surfactant binding, contributing to our understanding of viral behavior in the presence of surfactants. These findings have significant implications for improving water and wastewater treatment processes, as well as for the design of antiviral strategies targeting viral surface proteins.

The MIC is currently the best available parameter that can reflect the effectiveness of a chemical compound against a microorganism (Kowalska-Krochmal and Dudek-Wicher 2021). Compared to the gradient strip method, which is an alternative method to determine MIC, the dilution method in a liquid medium is recommended due to its accuracy, low cost, and specificity (Kowalska-Krochmal and Dudek-Wicher 2021). Our result indicated that the MICs for all surfactant and virus combinations were determined to be 1 mg/L, over a 60-minute exposure at room temperature followed by a 24-hour incubation period. This means that we need at least 1 mg/l of surfactants in a solution to inhibit virus growth. Since surfactants cannot be completely removed by the water treatment technologies, which leave residual concentration from 1-3 mg/L, it can be assumed that the residual surfactant in secondary effluent can inhibit virus growth (Giger et al. 1989, Bautista-Toledo et al. 2014). However, due to the low water temperature and the presence of other inorganic matters in the water, a longer interaction time between the virus and surfactants may be necessary to inhibit virus growth.

Several factors determine the efficiency of the MIC study. Based on our experimental results, the volume ratio of surfactant and viruses in each well is important. The exposure time and interaction temperature also influence the results. A limitation of this experiment is that the dilution series can only record the end-point analysis at the end of the 24-hour incubation time (Kowalska-Krochmal and Dudek-Wicher 2021). During this time, the PI dye in the solution may continuously affect the virus viability (Gawlitta et al. 2004). Additionally, there was not an effective way for us to remove the surfactants from the virus sample after the 1-hour interaction time. In this case, we only use more virus media to dilute the existing surfactants in each well. Despite dilution, the surfactants can still affect both cells and virus viability during the 24-hour incubation period. Finally, we found it challenging to differentiate between cell necrosis caused by PI dye, cell necrosis caused by surfactants, cell apoptosis, and cell pyroptosis caused by the virus using fluorescence images alone (Danthi 2016). Previous literature indicated that the SDS surfactant concentration above 0.4 mM influences DNase enzyme activities (OKABE et al. 1993). Our results indicated that both SDS and BAC at concentrations ranging from 1 mg/L to 100 mg/L do not interrupt DNase activity at room temperature for at least 15 mins. Additionally, qPCR quantification analysis tends to underestimate the quantity of degraded DNA/RNA (Gill et al. 2022).

## Conclusion

This study evaluates the interactions between ionic surfactants and enveloped and non-enveloped viruses in aqueous environments. The minimum inhibitory concentration (MIC) of surfactants required to inhibit virus infectivity and growth was determined under specific experimental conditions. The results support the following conclusions: The modeled isoelectric point (IEP) of proteins is generally higher than the experimentally determined IEP for both enveloped and non-enveloped viruses. Surfactants at different concentrations have varying effects on virus particle hydrated radii and zeta potential. Overall, the virus-hydrated diameters are larger than previously reported diameters in the literature without any surfactants present. Further, when surfactants are present, the hydrated diameter of both MHV and ADV were significantly lower. Specifically, the MHV hydrated diameter decreased from 150 ± 28 nm without surfactants to between 132 and 140 nm when either SDS or BAC were present. The ADV hydrated diameter was 142 ± 8 nm with no surfactants and decreased to between 109 and 116 nm when either surfactant was present. The IEP of both enveloped and non-enveloped viruses changes in the presence of surfactants. The experimental IEP results were lower than the modeling results based on amino acid structures alone. The IEP for mouse hepatitis virus (MHV) without surfactants was around 4.2, and the IEP of human Adenovirus (ADV) was 3.8. When the negatively charged sodium dodecyl sulfate (SDS) surfactant was present, the MHV and ADV IEP were reduced (i.e. 4.1 or lower for MHV and 3 or lower for ADV). Conversely, adding benzalkonium chloride (BAC), the cationic surfactant increases the IEP to 4.3 or higher for MHV. The MIC of surfactants for both MHV and ADV is 1 mg/L after a 60-minute interaction at room temperature. Molecular dynamics simulations revealed that SDS forms strong, localized interactions with viral surface proteins that markedly alter their electrostatic environment. In contrast, BAC engages only weakly and diffusely, indicating a minimal impact on protein structure and charge. This research will fill the gaps in environmental microbiology and nanoparticle studies and provide data for future research for modeling virus fate and transport in engineered treatment systems and the subsurface.

## Supplementary Material

xtaf011_Supplemental_Files
