# Supplementary material for: Ionic surfactants alter virus surface properties and electrostatic interactions in aqueous systems
Source: FEMS Microbes. 2025 Sep 11;6:xtaf011. doi: 10.1093/femsmc/xtaf011 (PMC12452283; doi:10.1093/femsmc/xtaf011)
Supplement: xtaf011_Supplemental_Files [file xtaf011_supplemental_files.zip › FEMSMC-2025-010.R1 one sentence summary.docx]

Ionic surfactants alter enveloped and non-enveloped viruses surface properties, isoelectric points and hydrated diameters through interactions with viral proteins.
