# Supplementary material for: Ionic surfactants alter virus surface properties and electrostatic interactions in aqueous systems
Source: FEMS Microbes. 2025 Sep 11;6:xtaf011. doi: 10.1093/femsmc/xtaf011 (PMC12452283; doi:10.1093/femsmc/xtaf011)
Supplement: xtaf011_Supplemental_Files [file xtaf011_supplemental_files.zip › Virus-surfactant ineraction paper, FEMS Microbe R1 supplemental info.docx]

Supplemental Information for

**Ionic Surfactants Alter Virus Surface Properties and Electrostatic Interactions in Aqueous Systems**

Makayla Loey^1^, Gabriel Costa Alverni Da Hora^2^, and Jennifer Weidhaas ^1^ *

^1^ University of Utah, Department of Civil and Environmental Engineering, 110 Central Campus Drive, Suite 2000, Salt Lake City, UT 84112,

^2^ University of Utah, Department of Chemistry, 315 1400 E, Salt Lake City, UT 84112,

* Corresponding author: Jennifer Weidhaas: [jennifer.weidhaas@utah.edu](mailto:jennifer.weidhaas@utah.edu), 801-585-1228

Table S1. Observed hydrated diameters for MHV and ADV at different pH and surfactant concentrations.

|  |  | Hydrated diameter, nm [average (standard deviation)] | | | | |
| --- | --- | --- | --- | --- | --- | --- |
| Virus | pH | No surfactant | 5 mg/L SDS | 10 mg/L SDS | 5 mg/L BAC | 10 mg/L BAC |
|  |  |  |  |  |  |  |
| MHV | 3 | 154 (13) | 145 (27) | 127 (37) | NA ^b^ | 151 |
| MHV | 4 | 148 (9) | 133 (29) | 154 (71) | 136 (16) | 143 (0.3) |
| MHV | 5 | 148 (10) | 132 (19) | 140 (3) | 156 (22) | 147 (6) |
| MHV | 6 | 150 (10) | 122 (29) | 137 (28 | NA | 170 |
| MHV | 7 | 148 (13) | 132 (25) | 148 (19) | 138 (31) | 124 (29) |
| MHV | 8 | 144 (4) | 120 (37) | 149 (12) | 145 (63) | 138 (41) |
| MHV | 9 | 158 (9) | 153 | NA | 138 (17) | 131 (39) |
|  |  |  |  |  |  |  |
| MHV | All^a^ | 150 (10) | 136 (28) # | 140 (37) | 139 (24) | 132 (31) |
|  |  |  |  |  |  |  |
| ADV | 3 | 135 (5) | 123 (24) | 120 (26) | 115 (16) | NA |
| ADV | 4 | 144 (4) | 123 (30) | 115 (27) | 130 (40) | NA |
| ADV | 5 | 145 | 123 (20) | 126 (18) | 110 (24) | NA |
| ADV | 6 | 143 | 110 (22) | 123 (20) | 107 (20) | NA |
| ADV | 7 | 144 (6) | 95 (22) * | 116 (28) | 119 (18) | NA |
| ADV | 8 | 149 (11) | 117 (21) * | 105 (15) * | 104 (16) | 117 (13) |
| ADV | 9 | 136 (9) | 101 (16) | 101 (14) * | 104 (21) | 116 (13) |
|  |  |  |  |  |  |  |
| ADV | All^a^ | 142 (8) | 112 (24) # | 110 (20) # | 109 (21) # | 116 (13) # |

^a^ All = observations at all pH levels; ^b^ NA = data not available, * Holm-Sidak comparison among mean hydrated diameter at a given pH showed a significance difference when surfactant is present at the given concentration, # Holm-Sidak comparison among mean hydrated diameter for all pH levels show a significant difference at the given surfactant concentration
