# Supplementary material for: Ionic surfactants alter virus surface properties and electrostatic interactions in aqueous systems
Source: FEMS Microbes. 2025 Sep 11;6:xtaf011. doi: 10.1093/femsmc/xtaf011 (PMC12452283; doi:10.1093/femsmc/xtaf011)
Supplement: xtaf011_Supplemental_Files [file xtaf011_supplemental_files.zip › FEMSMC-2025-010 supplementary data - reviews and author response.docx]

**Reviews of manuscript FEMSMC-2025-010**

**Reviewer 1**

**Recommendation: Minor Revision**

I find the manuscript interesting and worth publishing after the authors address the following concerns:

1. Probably, the most serious question is why the authors have chosen this particular spike protein for MD simulation? And, most importantly, why they claim that the results of simulation for this protein characterise the overall behaviour of the virus under the influence of the surfactants. If I misinterpret the authors' claims, please, make the text more clear what exactly the MD results mean in relation to the surfactant's influence on the virus as a whole.

2. Very puzzling to me were the data on the virus' hydrodynamic diameter becoming _smaller_ when the surfactants added. Some explanation of this is needed as, to me, this is rather counter intuitive. Maybe the surfactants somehow damage the lipid envelope in the case of MHV? Then, the MD results on the interaction between SDS and the spike protein, while important, could be not the only mechanisms of surfactant's action.

3. For simulation only 10 surfactant molecules were chosen. Why? Does this correspond to one of the experimental concentrations used in the study?

4. Figure 7 shows electrostatic potential and the authors claim that the surfactant makes it lower by comparing the colour. The colour does not look very different to me. More enhanced difference in the representation is needed (quantitatively the difference is present).

5. In relation to electrostatic properties of virus surface and their change by surfactants there is a series of relevant publications by Farafonov, Vodolazkaya and co-authors, which should be mentioned. Here are a few examples:

https://doi.org/10.1016/j.molliq.2023.122644

http://dx.doi.org/10.1021/acs.jpcb.2c04491

https://doi.org/10.1016/j.molliq.2020.112569

6. A few technical comments:

- In the table of Fig. 2 the units of the diameter are not specified.

- Line 345: two "0" concentrations are specified. I guess this is mistyping. But still it's unclear what "six steps" are used in the equilibration protocol? Those with the specified position restraints?

- Line 349: it's not clear what does "step 6.4" mean?

- Figure 5: it looks like two periodic copies of the simulation box are shown. Why?

- Figure 6 (and in the text): on the vertical axes of plots A-D "probability" is indicated, which can not be correct as the value of probability can't be great than 1. Perhaps, "frequency" was ment? Also, it's not clear what are "raw" and "normalized" probabilities? Probabilities are always "normalized" by definition.

- I couldn't make sense of the sentence on lines 757-758.

**Associate Editor Recommendation: Minor Revision**

thank you for submitting your manuscript to FEMS Microbes and apologies for the delay in reviewing your manuscript.

Your manuscript has now been reviewed by one external expert reviewer and assessed internally. Both assessments concur in considering your manuscript worthy of publication upon minor revision. Please see reviewer's comments below.

Please return the revised version of your manuscript within the time frame provided. If you cannot complete the required modifications within this time period, please contact me.

**EIC Decision: Minor Revision**

**Author's Response to Decision Letter for (FEMSMC-2025-010)**

We appreciate you providing the reviewer comments on our manuscript number FEMSMC-2025-010, entitled "Ionic Surfactants Alter Virus Surface Properties and Electrostatic Interactions in Aqueous Systems". We have addressed each comment point by point as shown below and have modified the manuscript and figures as necessary. We have attached a marked up version and a clean version of the manuscript. We now feel the manuscript is ready for publication and appreciate you handling this manuscript for us.

Kind regards,

Jennifer Weidhaas

Reviewer #1 comments

Comment: I find the manuscript interesting and worth publishing after the authors address the following concerns:

Response: Thank you for your careful review of the manuscript and insightful comments. We have addressed each comment below and revised the text in red-line strikeout as necessary in the manuscript. Revised figures have also been included as necessary in the new version of the manuscript.

Comment 1. Probably, the most serious question is why the authors have chosen this particular spike protein for MD simulation? And, most importantly, why they claim that the results of simulation for this protein characterise the overall behaviour of the virus under the influence of the surfactants. If I misinterpret the authors' claims, please, make the text more clear what exactly the MD results mean in relation to the surfactant's influence on the virus as a whole.

Response:

The spike protein from MHV-A59 (PDB ID: 1WDF) was selected for MD simulations due to its high-resolution structure, minimal missing residues (compared to others), and its relevance as a representative surface protein of the mouse hepatitis virus. This choice was made to ensure structural integrity and reliability in the simulation outcome, allowing comparison with the experiments.

Regarding the broader implications: the MD simulations do not claim to characterize the entire virus’s behavior but rather provide mechanistic insight into how surfactants may perturb key surface proteins. The spike protein is embedded in a lipid bilayer in the simulation, allowing us to observe surfactant-protein and surfactant-lipid interactions in a realistic membrane context. We revised the manuscript to clarify that the MD results are not a holistic model of the virus but a focused mechanistic study of surfactant effects on a critical viral surface protein. We inserted the text below in the Methods section:

“To investigate the dynamics of the MHV Spike Glycoprotein S and its interactions with a lipid bilayer and surfactants, molecular dynamics (MD) simulations were performed on two carefully designed systems. The spike protein from MHV-A59 (the RCSB PDB ID: 1WDF) (Xu et al., 2004) was selected due to its high-resolution structure, minimal missing residues, and its relevance as a representative surface protein of the virus. This choice ensures structural integrity and reliability in the simulation outcomes, enabling mechanistic comparisons with experimental observations. While the simulations do not model the entire virus, they provide atomistic insight into how surfactants perturb key viral components embedded in a realistic membrane context. The MHV Spike Glycoprotein S…”

Comment 2. Very puzzling to me were the data on the virus' hydrodynamic diameter becoming _smaller_ when the surfactants added. Some explanation of this is needed as, to me, this is rather counter intuitive. Maybe the surfactants somehow damage the lipid envelope in the case of MHV? Then, the MD results on the interaction between SDS and the spike protein, while important, could be not the only mechanisms of surfactant's action.

Response:

The text in the discussion has been revised to highlight some additional mechanisms whereby the surfactants could impact the virus proteins without necessarily dissolving the envelop or capsid entirely. Potential mechanisms for changing the hydrated diameters as measured by DLS include thinning of the envelop bilayers, stripping spike proteins or altering protein configurations. The following text was inserted in the discussion:

“Finally, these results could suggest that the low concentration surfactants are thinning or dissolving envelop bilayers (Becker et al., 1975, Negi et al., 2024), stripping the membrane of surface spike proteins (Simon et al., 2021), or altering the virus protein configuration to form more compact molten-globule states before unfolding at higher surfactant concentrations (Moosavi-Movahedi et al., 2003, Xu & Keiderling, 2004).”

Comment 3. For simulation only 10 surfactant molecules were chosen. Why? Does this correspond to one of the experimental concentrations used in the study?

Response:

The choice of ten surfactant molecules per simulation system was deliberate, balancing computational tractability with the need to observe specific molecular interactions. This number allows for detailed tracking of surfactant-protein, surfactant-lipid, and surfactant-surfactant interactions with high spatial and temporal resolution over the course of a 1 μs simulation. While this does not directly replicate the experimental concentrations (5 and 10 mg/L), the simulated concentrations were approximately 17.3 mg/L for SDS and 27.4 mg/L for BAC, corresponding to ~8.6 mM in both cases. These values (higher than the experimental conditions) were chosen to enhance the likelihood of observing multiple interaction events across distinct protein regions within a limited simulation timescale. This approach is common in mechanistic MD studies, where the emphasis is on interaction specificity and atomistic insight rather than ensemble averaging or bulk behavior. We added to the manuscript:

“To facilitate the observation of multiple interaction events within a feasible simulation timescale, surfactants were introduced at concentrations higher (17.3 mg/L for SDS and 27.4 mg/L for BAC) than those used experimentally. This design choice enabled the identification of distinct binding regions and interaction patterns without compromising the atomistic resolution of the system.”

Comment 4. Figure 7 shows electrostatic potential and the authors claim that the surfactant makes it lower by comparing the colour. The colour does not look very different to me. More enhanced difference in the representation is needed (quantitatively the difference is present).

Response:

Thank you. The visual representation could be improved. We have chosen the default options of the electrostatic potential maps in the first version. Now, we have regenerated the APBS maps with enhanced contrast and consistent color scales to better highlight the neutralization effects, especially in the regions of surfactant accumulation. We also included the electrostatic energy values directly in the figure caption to reinforce the quantitative differences:

“Figure 7. Electrostatic potential maps of the MHV Spike Glycoprotein S were obtained using the APBS server. Side and bottom views are shown for the protein alone, with SDS, and with BAC. Regions of surfactant interaction (top and bottom) exhibit visible charge neutralization, transitioning from red (positive) to white or pale blue (neutral/negative). SDS induces a stronger electrostatic shift, consistent with its lower total electrostatic energy (1.96×10⁵ kJ/mol) compared to BAC (2.20×10⁵ kJ/mol) and the unbound protein (2.39×10⁵ kJ/mol).”

Comment 5. In relation to electrostatic properties of virus surface and their change by surfactants there is a series of relevant publications by Farafonov, Vodolazkaya and co-authors, which should be mentioned. Here are a few examples:

https://doi.org/10.1016/j.molliq.2023.122644

http://dx.doi.org/10.1021/acs.jpcb.2c04491

https://doi.org/10.1016/j.molliq.2020.112569

Response:

Thank you for pointing out these references. We concur that two of the references are relevant and highlight the electrostatic properties of virus surfaces as well as the influence of surfactants and NaCl on apparent hydrated diameters. The text in the discussion has been revised to add the following text:

“As others have reported that the buffer concentration can influence the apparent hydrated diameter, (i.e., increasing NaCl concentrations decreased apparent hydrated diameter from 33 nm to 30 nm (Vodolazkaya et al., 2022)) we were careful in our studies to use one ionic strength solution for all experiments.”

And

“Decreases in hydrodynamic diameter of MS2 particles with the addition of SDS and Triton X-100 was also observed by others (Vodolazkaya et al., 2023) although the decrease in diameters decreased by only 2 to 4 nm (i.e., 35 nm to 33 nm with SDS and 34 to 30 nm with Triton X-100).”

Comment 6. A few technical comments:

- In the table of Fig. 2 the units of the diameter are not specified.

Response:

Thank you we have added the units of “nm” to the table.

Comment - Line 345: two "0" concentrations are specified. I guess this is mistyping. But still it's unclear what "six steps" are used in the equilibration protocol? Those with the specified position restraints?

Response:

We agree that this paragraph needs clarification. The “six steps” refer to the CHARMM-GUI equilibration protocol, which gradually relaxes position and dihedral restraints of each component to stabilize the system. The two “0” concentrations were a typographical error and will be corrected. We rewrote the paragraph to explicitly list the six steps, including the restraint values and timestep changes, and explain that this protocol ensures a smooth transition from a highly restrained system to a fully relaxed one suitable for production MD:

“The timestep was set to 1 fs for the first three steps and increased to 2 fs for the final three. Position restraints on the protein backbone, sidechains, and lipids were progressively reduced across the six steps (from 4000 2000 1000 500 200 50 kJ/mol/nm² for the backbone; 2000 1000 500 200 50 0 kJ/mol/nm² for sidechains; and 1000 400 400 200 40 0 kJ/mol/nm² for lipids). Dihedral restraints were similarly relaxed (1000 400 200 200 100 0 kJ/mol/nm²) to allow for sidechain and secondary structure relaxation.”

Comment - Line 349: it's not clear what does "step 6.4" mean?

Response:

Thank you for pointing this out. It refers to the fourth step in the six-step equilibration protocol, where pressure coupling is introduced. We have revised the text for clarity (see previous Response).

Comment - Figure 5: it looks like two periodic copies of the simulation box are shown. Why?

Response:

The intent was to better illustrate surfactant distribution across the membrane, but we agree that showing two periodic images may be confusing. We revised the figure to show only one simulation box.

Comment - Figure 6 (and in the text): on the vertical axes of plots A-D "probability" is indicated, which can not be correct as the value of probability can't be great than 1. Perhaps, "frequency" was meant? Also, it's not clear what are "raw" and "normalized" probabilities? Probabilities are always "normalized" by definition.

Response:

We thank the reviewer for this insightful observation. The values shown in Figure 6A–D represent binding probabilities expressed as percentages, calculated as the fraction of simulation frames in which a given residue is in contact (within a 4.0 Å cutoff) with any surfactant atom, multiplied by 100. The y-axis label should have included the “%” symbol to clarify this, and we have corrected the figure accordingly. Regarding the distinction between “raw” and “normalized” probabilities: SDS contains four sulfate oxygens capable of forming electrostatic contacts, whereas BAC has only one charged nitrogen. To enable fair comparison across surfactants with differing numbers of interaction sites, we normalized the SDS binding probabilities by dividing the raw values by four, yielding an effective per-site probability. BAC’s raw and normalized values are identical due to its single charged site. We have revised the corresponding text in the Results section to clarify these definitions and ensure accurate interpretation.

“To account for the multivalency of surfactants, we distinguish between raw and normalized probabilities. SDS possesses four sulfate oxygens capable of forming electrostatic contacts, whereas BAC contains only a single charged nitrogen. Therefore, raw binding probabilities for SDS were divided by four to yield an effective per-site probability, allowing a more fair comparison across surfactants.”

Comment - I couldn't make sense of the sentence on lines 757-758.

Response: The conclusion has been modified to clarify the ranges of hydrated diameters with and without surfactants. The following text is now present in the conclusion:

“Overall, the virus-hydrated diameters are larger than previously reported diameters in the literature without any surfactants present. Further, when surfactants are present, the hydrated diameter of both MHV and ADV were significantly lower. Specifically, the MHV hydrated diameter decreased from 150 ± 28 nm without surfactants to between 132 and 140 nm when either SDS or BAC were present. The ADV hydrated diameter was 142 ± 8 nm with no surfactants and decreased to between 109 and 116 nm when either surfactant was present.”

**Reviews of manuscript FEMSMC-2025-010.r1**

**Associate Editor Recommendation: Accept**

**EIC Decision: Accept**
